# Effectiveness and Safety of the MVA–BN Vaccine against Mpox in At-Risk Individuals in the United States (USMVAc)

**DOI:** 10.3390/vaccines12060651

**Published:** 2024-06-11

**Authors:** Soowoo Back, Bethany Knox, Ciara Coakley, Nicolas Deltour, Emmanuelle Jacquot, Hanaya Raad, Elizabeth M. Garry

**Affiliations:** 1 Aetion, Inc., New York, NY 10001, USA; 2 Aetion Iberia, S.L., 08037 Barcelona, Spain

**Keywords:** vaccine effectiveness, vaccine safety, MVA–BN, mpox (monkeypox), real-world evidence (RWE)

## Abstract

The mpox 2022 outbreak was declared a public health emergency in July 2022. In August 2022, the MVA–BN vaccine received emergency use authorization in the United States (US) to target at-risk groups. This study (EUPAS104386) used HealthVerity’s administrative US healthcare data to generate real-world evidence for MVA–BN vaccine effectiveness and safety to prevent mpox disease in men who have sex with men (MSM) and transgender women, the most affected population during the 2022 mpox outbreak. Fully vaccinated subjects (two doses ≥ 28 days apart) were initially matched with five unvaccinated subjects on calendar date, age, US region, and insurance type. Subjects were followed from index date (14 days after the second dose) until death or data end to ascertain mpox occurrence. After propensity score adjustment, the MVA–BN vaccine effectiveness against mpox disease was 89% (95% CI: 12%, 99%) among those fully vaccinated; attenuated to 64% (95% CI: 40%, 78%) among those with any dose and 70% (95% CI: 44%, 84%) for those with only a single dose. One pericarditis adverse event of special interest was observed when the risk window was extended to 28 days. These results contribute to the totality of evidence supporting the favorable benefit/risk profile of the MVA–BN vaccine.

## 1. Introduction

Mpox disease (previously known as ‘monkeypox’) is caused by the zoonotic, human *monkeypox virus* (MPXV) and is akin to the smallpox virus. Historically, this clade II MPXV was endemic to West Africa and self-limited in human-to-human transmission. Previously reported mpox cases in the United States (US) were associated with international travel to endemic regions. However, in 2022, like other historically non-endemic countries, the US experienced 30,344 cases and 42 deaths, approximately one-third of the total 2022 outbreak global cases [1,2,3]. The majority of the US cases have occurred in persons who identify as men or transgendered persons who have sex with men (MSM). Approximately 40% of these MSM have concomitant human immunodeficiency virus (HIV) infection, putting them at increased risk of severe disease [4,5,6]. In May 2023, it was declared that the multi-country mpox outbreak was no longer a Public Health Emergency of International Concern [7]. However, the more than 91,000 cases of human-to-human transmission of mpox since 2022, the rise in infections among the unvaccinated that prompted a health alert in New York City [8], and a recent 2024 resurgence in the Democratic Republic of the Congo (DRC) demonstrate a continued need for preventive vaccination efforts. Of increasing concern is the clade I MPXV endemic to the DRC, as nearly 10,000 cases were reported between January 2023 and April 2024. Clade I has a more severe prognosis than clade II, with a 4.9% case fatality rate [9,10]. Though no clade I MPXV cases have been detected in the US, the Centers for Disease Control and Prevention (CDC) emphasizes the need for public health preparedness.

The Modified Vaccinia Ankara-Bavarian Nordic (MVA–BN) vaccine is a third-generation smallpox vaccine approved by the US Food and Drug Administration [11] in 2019 for subcutaneous administration for the prevention of smallpox and mpox disease. The mpox vaccine may be administered within 4 to 14 days after contact with an infected person per recommendations of the CDC to prevent the onset of the disease [12,13]. A full vaccination regimen includes two separate doses administered four weeks (28 days) apart [14]. The vaccination is considered effective 14 days after the second vaccine dose, though there is evidence to suggest some protection after the first dose [15,16,17]. MVA–BN may also be used as a booster vaccination (a single dose) in individuals previously vaccinated against smallpox, although there is inadequate data to determine the appropriate timing of booster doses [15,16,17,18].

In June 2022, the US national mpox vaccine strategy was first announced, and in July 2022, the World Health Organization [19] declared the mpox outbreak a public health emergency [19,20]. Shortly after, the European Medicines Agency approved the extension of indication for the MVA–BN vaccine to include the prevention of mpox and vaccinia-caused disease for use in the European Union (EU) [21]. Similarly, in the US, the Food and Drug Administration issued an emergency use authorization in August 2022 with the indication to prevent mpox in adults determined to be at high risk for mpox infection [11]. This triggered the need to generate real-world vaccine effectiveness and safety evidence in this population.

This retrospective, observational study of the MVA–BN vaccine (USMVAc; EUPAS104386) leverages administrative US healthcare data to evaluate effectiveness and safety among vaccinated MSM and transgender women, and it is part of a larger research program that includes two German prospective and retrospective studies (SEMVAc and TEMVAc; EUPAS50093) to support decision making in the EU regarding the benefit/risk profile of the MVA–BN vaccine.

## 2. Materials and Methods

### 2.1. Study Design and Data Source

This retrospective, observational comparative cohort study used adjudicated medical and prescription claims, laboratory data, and beneficiary demographic information among subjects with available information on vaccination status and diagnosis of mpox between 1 April 2021 and 31 December 2022 from a de-identified administrative US healthcare dataset curated by HealthVerity from their healthcare ecosystem that includes more than 200 million individuals from all US states insured under commercial, Medicare, or Medicaid plans, and/or served by providers participating in several large US medical and pharmacy insurance claims submission systems.

A set of initial inclusion criteria was applied by HealthVerity to create a therapeutic area-specific subset of those most ‘at-risk’ of mpox in the US. The subset criteria to identify the MSM and transgender women population were established using a proxy definition of MSM grounded in previous and ongoing research on mpox disease [22,23,24]. This required all subjects to fulfill at least 1 of the following criteria, in addition to being male according to sex assigned at birth and ≥18 years of age: (1) Presence of a diagnosis code related to High-Risk Sexual Behaviour; (2) presence of a diagnosis code indicating HIV infection; or (3) presence of a drug or procedure code indicating HIV pre-exposure prophylaxis (PrEP) among those without a diagnosis code indicating history of substance use disorder within the study period. (see Supplemental Material Table S1 for additional details)

### 2.2. Study Population and Patient Selection Criteria

The study population selected subjects from the ‘at risk’ data subset between 1 August 2022 (MVA–BN US emergency use authorization date) and 30 September 2022 to capture the peak of the outbreak in the US and allow for a minimum of 3 months of follow-up to evaluate vaccine effectiveness. For the primary vaccine effectiveness assessment, subjects were included in the exposure group if they had a second record of MVA–BN vaccine administration at least 28 days after the 1st dose and were continuously enrolled until the 14th day after their second vaccination, which served as the assigned index date.

Given the absence of an active comparator, for each vaccinated subject, coarsened exact matching (CEM) was used to select 5 similar unvaccinated subjects and assign them a matched index date. Subjects were included in the comparator group if they were matched to an exposed subject on calendar date, age group (18–25, 26–35, 36–45, >45), US region (Northeast, Midwest, South, West), and insurance type (commercial, non-commercial) and had no record of MVA–BN vaccine administration before or on the matched index date.

Additionally, subjects were excluded from both groups if they had a missing value for age, region, or sex on the index date or did not have at least 1 year of continuous enrollment (with 45 days of allowable gap) prior to and including the index date. Further, to capture the incident cases of mpox, subjects were excluded if they had a prior positive result of an orthopox PCR test or a mpox diagnosis code with at least 1 prescription of tecovirimat, vaccinia immune globulin intravenous, cidofovir, or brincidofovir (indicated for the treatment of smallpox and received expanded access for treatment of mpox during the outbreak).

### 2.3. Outcome Definitions and Follow-Up

The primary outcome of mpox disease was defined as the occurrence of a positive orthopoxvirus PCR laboratory test result indicating mpox or a diagnosis of mpox using ICD-10-CM diagnosis code B04 (any position, occurring in an inpatient or outpatient medical claim) or PCR result of “Detected”. Follow-up began on the index date (14 days after the 2nd MVA–BN dose) and concluded at the earliest occurrence of any of the following events: occurrence of mpox, disenrollment, death, or the end of data (31 December 2022). Comparator unvaccinated subjects were also censored upon administration of an MVA–BN vaccine dose if later vaccinated after the matched index date.

The secondary safety outcomes were selected in line with the outcomes classified as important potential risks for MVA–BN in the EU Risk Management Plan [21] and identified as Adverse Events of Special Interest (AESIs): myocarditis, pericarditis, encephalitis, anaphylaxis. These events were defined by ICD-10-CM codes and selected based on clinical relevance. Each safety outcome required a specified risk window. The primary risk windows after vaccination were 1 day for anaphylaxis, 14 days for myocarditis and pericarditis, and 28 days for encephalitis, and these were extended to 3, 28, and 42 days, respectively, in sensitivity analyses. Follow-up ended on the first occurrence within the specific risk window for each event or disenrollment, death, end of data (31 December 2022), or the end of the corresponding risk window.

### 2.4. Statistical Analyses

Demographic variables were assessed on the index date, and clinical variables were assessed during the baseline period, defined as the start of available data (as early as 1 April 2021) until 1 day before the index date (ranging from 1 August to 30 September 2022). After patient selection and CEM, 1:1 propensity score (PS) matching was attempted to control for confounding by measured covariates between vaccinated and unvaccinated groups. The PS was estimated using multivariable logistic regression based on the probability of MVA–BN exposure conditional on the following covariates: age, region, insurance provider, race, HIV infection, PrEP use during baseline, history of sexually transmitted infections (STI), evidence of autoimmune conditions or non-HIV related immunocompromised conditions, history of select comorbidities, and prior AESIs (for the assessment of safety outcomes only).

The balance of covariates was based on comparing absolute standardized difference (ASD) for each measured covariate and assessed before and after PS-based adjustment. Groups were considered balanced if more than 90% of the covariates had an ASD < 0.10 (+/−0.04). Subjects with missing values were retained in the analytic cohort, and missing values were reported.

For the assessment of the effectiveness of full vaccination, Standardized Mortality/Morbidity Ratio (SMR) weighting was used as an alternative PS adjustment method since PS matching excluded > 10% of vaccinated subjects from the analytic cohort. This approach applied a weight of 1 to vaccinated patients and a weight of PS/(1 − PS) to unvaccinated comparator subjects. Extreme weights above the 99th percentile of the weight distribution were truncated. Weighted risk differences and vaccine effectiveness percentages in the vaccinated exposed group and the unvaccinated comparator group were reported along with corresponding 95% confidence intervals (CI).

### 2.5. Secondary and Sensitivity Analyses

For the assessment of the secondary safety outcomes, subjects were included on the index date in the exposure group if they had any record of at least one dose of the MVA–BN vaccine, and all inclusion/exclusion criteria were previously met. PS matching (with a ratio of 1:1) was used to control for measured confounding. The safety of MVA–BN was determined based on the count of patients with a myocarditis, pericarditis, encephalitis, or anaphylaxis event in the vaccinated group versus unvaccinated subjects, and risk differences and 95% confidence intervals were reported if there were any reportable events.

For a better understanding of vaccination among MSM and transgender women, who were the most affected population in the US, vaccine effectiveness was assessed within subgroups of patients with and without evidence of HIV infection and treatment status (antiretroviral or PrEP use). Sensitivity analyses were conducted using a subset of the overall vaccinated subjects, and PS matched unvaccinated comparator subjects who received ‘any dose’ (≥1), those who received ‘single dose’ (1 dose only), and those who received ‘single dose’ assumed to have a prior smallpox vaccination, inferred by subjects >50 years. SMR weighting was re-applied within HIV and treatment subgroups. PS matching (with a ratio of 1:1) was used to control for measured confounding.

Analyses were performed using Aetion^®^ Substantiate (2023) software for real-world data analysis version 4.88, which is validated for a range of studies [25] and R version 3.4.2.

## 3. Results

### 3.1. Vaccine Effectiveness

**Figure 1:**
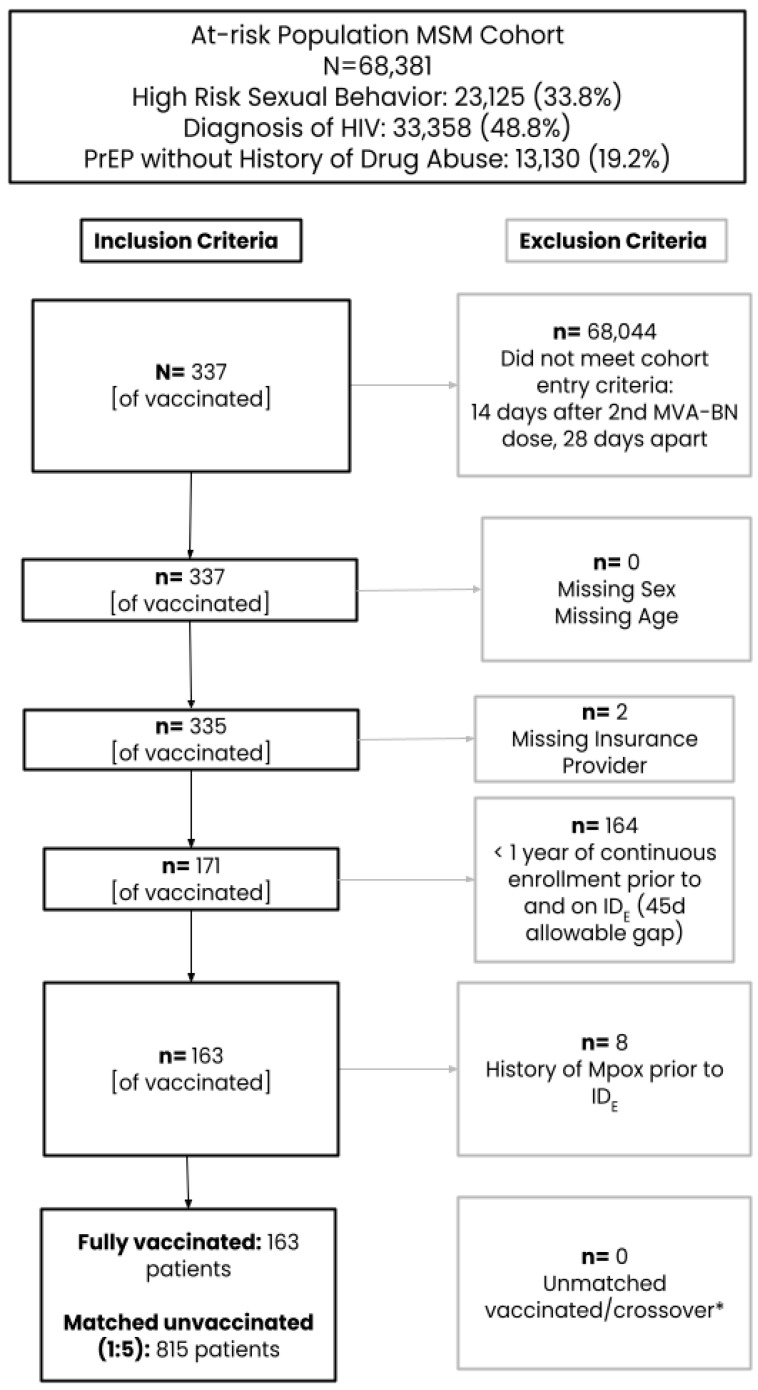
Cohort attrition. * Exposed subjects are matched to unexposed subjects on calendar date, age, region, and insurance provider in chronological order The matching was done without replacement (each patient can only be sampled once and contribute to the treated or untreated group, depending on whichever group that they are selected to first, therefore some patients may be “crossover” patients, or patients who were selected as referent unexposed patients at the time of selection before they could be selected as exposed patients.

After the application of inclusion/exclusion criteria and CEM, the initial primary cohort for the assessment of vaccine effectiveness included 163 fully vaccinated subjects (received two doses ≥ 28 days apart) matched to 815 unvaccinated subjects (Figure 1). Only 6.1% of subjects were aged 18–25 years, while the remaining subjects were similarly split across the age categories of 26–35 (31.9%), 36–45 (27.0%), and at least 45 (35.0%) years (Table 1). The majority of the study population was commercially insured and largely represented the Western US region. Evidence of an STI during baseline was low but similar between groups (~5%). When fully vaccinated subjects were compared to the unvaccinated comparator group, more were missing capture of a race category (40.5% vs. 7.4%), people living with HIV (PLWHIV) (65.0% vs. 56.4%) or have an autoimmune disorder (28.2% vs. 26.0%) or PrEP use (29.4% vs. 22.0%), while fewer subjects were white (30.7% vs. 45.8%) and had other comorbidities (27.6% vs. 32.4%). After SMR weighting was applied, the 163 fully vaccinated subjects were compared to a weighted pseudo-population of 159.85 with an effective sample size of 303. All covariates included in the PS model were balanced between vaccinated and unvaccinated groups with ASDs ≤ 0.10.

In the subgroups of the fully vaccinated and unvaccinated comparator subjects stratified by evidence of HIV infection and antiretroviral treatment (ARV), imbalances prior to PS adjustment were observed; however, more than 90% of the measured covariates had an ASD < 0.10 [+/−0.04]) after PS weighting, which was considered balanced per protocol (Supplemental Tables S2 and S3). All fully vaccinated subjects who were PLWHIV were under ARV treatment and accounted for nearly two-thirds (63.8%) of the fully vaccinated overall cohort. Thus, there were no fully vaccinated subjects who were PLWHIV without ARV treatment, and the remaining subgroups of subjects who were HIV-negative with and without PrEP use were quite small.

Compared to unvaccinated subjects, there was a decreased risk of mpox disease among those in the overall cohort of fully vaccinated subjects after PS adjustment (risk difference per 100,000 persons, −4931.64, 95% CI: −8833.30, −1029.98), resulting in a vaccine effectiveness of 89% (95% CI: 12%, 99%) to prevent mpox (Table 2). While results in the subgroup of PLWHIV under ARV treatment (that made up the majority of the overall cohort) suggested similar vaccine effectiveness after PS adjustment, the confidence intervals were wide and inclusive of 0% (71%, 95% CI: −162%, 97%). Due to the small sample sizes of subjects vaccinated and unvaccinated in each subgroup without HIV, there were no reportable differences in vaccine effectiveness with respect to mpox between users and non-users of PrEP.

In the sensitivity analyses, vaccine effectiveness was attenuated to 64% (95% CI: 40%, 78%) among those with any dose and 70% (95% CI: 44%, 84%) among those with only a single dose; there was no evidence of vaccine effectiveness observed for single dose vaccination among those aged >50 years assumed to have a prior vaccine for smallpox (Table 3).

### 3.2. Vaccine Safety

The initial secondary cohort for the assessment of safety included 947 subjects receiving any dose matched to 4735 unvaccinated subjects via CEM (Table 4). Using a 1:1 PS matching process, the final PS model, including all covariates except for history of myocarditis and history of encephalitis, was selected. History of myocarditis and history of encephalitis were removed due to having no patients in the exposed or comparator group, leading to a violation of positivity for those covariates. All covariates included in the PS model were determined to be effectively balanced. Overall, there were <5 subjects with a history of AESIs during the baseline period in either group.

There were no AESI events among those who received at least one dose of the MVA–BN vaccine and their matched comparator subjects within the specified risk windows for each safety outcome (Table 5). After expanding the risk window to 28 days for pericarditis, one pericarditis event was identified in the vaccinated subjects after PS adjustment (risk per 100,000 persons, 109.29, 95% CI: 15.38, 776.69).

## 4. Discussion

In this study using administrative US healthcare data, high vaccine effectiveness of 89% was observed in the primary analysis among a small cohort of subjects who received full vaccination with two doses relative to a matched sample of unvaccinated subjects considered to be at a similar risk. While the interpretation of these results may be limited by the small sample size, they are consistent with existing US studies of subjects fully vaccinated, including two case-control studies that reported vaccine effectiveness of 66% using a nationwide Epic electronic health record database and 86% using health department case registries from 12 states, and a study conducted in New York State that reported 76% vaccine effectiveness in men diagnosed with mpox compared to negative controls with rectal gonorrhea or primary syphilis [13,15,26,27], in addition to other recent systematic reviews and meta-analysis of current literature on real-world evidence of MVA–BN vaccine prevention of mpox that reported vaccine effectiveness ranging from 66% to 90% [28,29]. Further, these results align with recent CDC Morbidity and Mortality Weekly Reports that show that breakthrough mpox infections in the US after two doses of the MVA–BN vaccine are rare [30,31].

In the sensitivity analyses where the sample size was larger, vaccine effectiveness among subjects with any dose and among subjects with a single dose was 64% and 70%, respectively, which was lower than but consistent with previous studies that reported protection against mpox infection 14 days after the first dose. A study in the United Kingdom reported a single-dose vaccine effectiveness of 78%, similar to a study from Israel, which reported 86% among high-risk populations [17,18]. A key difference in our analyses is that our sensitivity analyses of vaccine effectiveness of single-dose MVA–BN vaccination began follow-up starting 1 day after vaccination, prior to the required 14 days to achieve the maximum protective effect. There were 3 cases of mpox in the vaccinated group versus 15 cases in the unvaccinated within the 14-day window after vaccination with the first dose.

No confirmed cases of myocarditis, pericarditis, or encephalitis were observed in completed preclinical and clinical trials of MVA–BN [18,32]; similarly, no cases of myocarditis following 7 or 30 days after MVA–BN vaccination were observed in a Canadian prospective safety surveillance study [33] and surveillance data from the US Vaccine Adverse Event Reporting System did not identify any unexpected safety concerns [34]. As in previous studies, no safety events were observed in this study among those receiving any dose of the MVA–BN vaccine during the primary risk windows. There was, however, one pericarditis event in the vaccinated group when the risk window was extended to 28 days.

There are several considerations when interpreting the results of this study. As healthcare claims originate for insurance billing purposes, there is a financial incentive to confirm accuracy. However, the risk of misclassification is inherent in all claims-based data due to provider coding practices (e.g., using a diagnosis code as a rule-out criterion) or coding errors. As with most secondary data sources, HealthVerity does not have available information on sexual orientation and gender identity and does not contain clinical notes or other documentation that could be utilized to validate the MSM and transgender women algorithm used for selection into the study cohort. Misclassification of MSM and transgender women is, therefore, possible and differentially more likely among unvaccinated subjects since we can assume that vaccinated subjects were indicated to receive the vaccine given their risk profile. If there are men in the unvaccinated group who are not MSM or transgender women, the risk of mpox may be underreported as these subjects would not have the same risk profile as those vaccinated, thereby overestimating vaccine effectiveness. However, the proxy definition of MSM and transgender women used was based on previous studies of mpox disease in the MSM population [16,17,35,36] and selected subjects presumed to be MSM or transgendered women based on high-risk criteria. While the lack of gender identity and sexual behavior is a limitation of this study, the broader program includes in-progress prospective and retrospective data collection studies in Germany in which subjects self-report gender identity and sexual behavior in the context of mpox to complement this work.

Further, the precision of estimates was limited by the sample size of subjects who met the cohort eligibility requirements during the study period and had complete data captured on their covariates in the HealthVerity dataset and the short follow-up available at the time of acquiring the data. While the interpretation of results is limited by our small sample size and number of patients experiencing the outcome, our results provide important information that contributes to the totality of evidence of the effectiveness and safety of the MVA–BN vaccine. Requiring a second MVA–BN dose at least 28 days apart and continuous enrollment at least 14 days after the second dose during the study period excluded 99.5% of subjects, likely due to accessibility of the vaccine. In the US, the mpox vaccine was available at no or minimal cost to recipients from the local health department or, in large cities, in public health clinics, hospitals, or at large social gatherings or venues [37]. These facilities and services may not always bill or submit to insurance for reimbursement, which may lead to underreporting of vaccination status in claims data and skew our study population towards those who are more frequently engaged in the healthcare system.

Lastly, the potential for residual confounding exists in observational cohort analyses. However, we employed good research practices using CEM to control for time-related biases, which are assumed to be strong given the evolving healthcare landscape of the mpox epidemic, and PS adjustment to control for confounding due to measured covariates. Although factors causing residual confounding may manifest after the index if there are changes in a subject’s behavior (e.g., a sudden increase in sexual partners), this study does not account for factors after the index date to avoid potential bias that may be induced by conditioning on mediating variables in the causal pathway, or on colliders.

## 5. Conclusions

The results from this retrospective, comparative real-world study add to the totality of evidence of the effectiveness and safety of the MVA–BN vaccine despite imprecise estimates due to the small sample size, noting that the greatest effectiveness was among those fully vaccinated with the two recommended doses. While mpox is no longer considered a public health emergency of international concern, cases continue to be reported. An accumulation of evidence from studies like this has the potential to help those coping with their own risk perceptions and vaccination decisions in non-endemic countries like the US, as well as broader populations in endemic countries like the DRC. Further studies with increased sample size and longer follow-up, including forthcoming results from the broader program of three studies that include prospective and retrospective data collection in Germany (SEMVAc and TEMVAc; EUPAS50093), are warranted for a more comprehensive assessment of the MVA–BN benefit/risk profile.

## Figures and Tables

**Table 1:** Baseline characteristics of adult men who have sex with men (MSM) and transgender women prior to and after SMR weighting.

Variable	Fully Vaccinated	Comparator Unvaccinated Prior to SMR Weighting ^a^	ASD ^b^ (Unweighted)	Comparator Unvaccinated after SMR Weighting ^c^	ASD ^b^ (Weighted)
Number of subjects	163	815	-	303	-
Sum of weights	NA	NA	-	159.85	-
Age on index date					
18–25 years	10 (6.1%)	50 (6.1%)	0.000	9.5 (5.9%)	0.009
26–35 years	52 (31.9%)	260 (31.9%)	0.000	53.9 (33.7%)	0.039
36–45 years	44 (27.0%)	220 (27.0%)	0.000	40.4 (25.3%)	0.040
>45 years	57 (35.0%)	285 (35.0%)	0.000	56.1 (35.1%)	0.002
US Region on index date					
Northeast	26 (16.0%)	130 (16.0%)	0.000	24.1 (15.0%)	0.025
Midwest	25 (15.3%)	125 (15.3%)	0.000	22.7 (14.2%)	0.033
South	39 (23.9%)	195 (23.9%)	0.000	35.9 (22.4%)	0.035
West	73 (44.8%)	365 (44.8%)	0.000	77.3 (48.3%)	0.071
Insurance Provider on index date					
Commercial	94 (57.7%)	470 (57.7%)	0.000	87.1 (54.5%)	0.065
Non-Commercial	69 (42.3%)	345 (42.3%)	0.000	72.8 (45.5%)	0.065
Race/Ethnicity on index date					
White	50 (30.7%)	373 (45.8%)	0.314	49.7 (31.1%)	0.010
Race other than white ^d^	47 (28.8%)	382 (46.9%)	0.379	47.0 (29.4%)	0.012
Missing	66 (40.5%)	60 (7.4%)	0.843	63.1 (39.5%)	0.020
HIV+ during baseline	106 (65.0%)	460 (56.4%)	0.177	107.0 (67.0%)	0.041
PrEP use during baseline	48 (29.4%)	179 (22.0%)	0.172	45.8 (28.6%)	0.018
Time from first PrEP in baseline to index date					
0–6 months	19 (11.7%)	52 (6.4%)	0.185	15.0 (9.4%)	0.073
6 months–1 year	12 (7.4%)	57 (7.0%)	0.014	12.5 (7.8%)	0.017
≥1 year	17 (10.4%)	70 (8.6%)	0.063	18.2 (11.4%)	0.031
No PrEP in baseline	115 (70.6%)	636 (78.0%)	0.172	114.1 (71.4%)	0.018
STI during baseline ^e^	8 (4.9%)	40 (4.9%)	0.000	9.5 (5.9%)	0.046
Evidence of (non-HIV) immunocompromised conditions during baseline ^f^	46 (28.2%)	212 (26.0%)	0.050	44.3 (27.7%)	0.011
Comorbidities during baseline ^g^	45 (27.6%)	264 (32.4%)	0.105	44.7 (28.0%)	0.009

HIV = human immunodeficiency virus; NA = Not applicable as prior to application of SMR weights; PrEP = pre-exposure prophylaxis; US = United States. Index date was the 14th day after the second dose. Baseline period = Start of data to 1 day before Index Date. ^a.^ Subjects in the vaccinated group were coarsened exact matched with 5 subjects who were unvaccinated at the time of match. ^b.^ Absolute standardized differences (ASDs) ≥ 0.10 are depicted in bold text. ^c.^ Although balance was achieved using 1:1 PS matching, 18 fully vaccinated subjects (>10%) were dropped; thus, SMR weighting was applied assigning vaccinated patients a weight of 1 and unvaccinated subjects a weight of PS/(1 − PS) to create a pseudo-population of weighted unvaccinated comparator subjects. ^d.^ Includes subjects with a race recorded as Black, Hispanic, Asian, or Other. ^e.^ Sexually transmitted infections (STI) were chlamydia, gonorrhea, syphilis, hepatitis B. ^f.^ Autoimmune disease, immunodeficiency, inhaled corticosteroids, immunomodulating medications, and immunotherapy. ^g.^ Cancer, diabetes, atopic dermatitis (neurodermatitis), and rheumatological, hematological, chronic cardiovascular, chronic lung, chronic kidney, and chronic liver disease.

**Table 2:** Vaccine effectiveness in adult men who have sex with men (MSM) and transgender women, primary overall cohort, and secondary subgroups based on HIV status and treatment ^a^.

	Total No. of Subjects	Sum of Weights	No. of Subjects with an Mpox Event ^b^	Risk per 100,000 Persons (95% CI)		Risk Difference per 100,000 Persons (95% CI)		Vaccine Effectiveness (95% CI)	
				Crude	Adjusted ^c^	Crude	Adjusted ^c^	Crude	Adjusted ^c^
Primary Analysis: Overall Cohort									
Fully Vaccinated	163	163.00	1	613.50 (85.90, 4381.64)	613.50 (85.90, 4381.64)	−858.90 (−2321.85, 604.06)	−4931.64 (−8833.30, −1029.98)	58% (−222%, 95%)	89% (12%, 99%)
Comparator Unvaccinated	813	159.85	12	1472.4 (760.81, 2571.97)	5545.14 (2839.90, 10,827.36)	reference	reference	reference	reference
Subgroup: HIV+ and Antiretroviral Use									
Fully Vaccinated	104	104.00	1	961.54 (134.17, 6891.13)	961.54 (134.17, 6891.13)	−1253.65 (−3750.87, 1243.57)	−2303.76 (−5920.90, 1313.38)	57% (−255%, 95%)	71% (−162%, 97%)
Comparator Unvaccinated	134	107.50	7	2215.19 (890.62, 4564.14)	3265.30 (1270.67, 8391.00)	reference	reference	reference	reference
Subgroup: HIV+ and No Antiretroviral Use									
Fully Vaccinated	0	0	0	-	-	-	-	-	-
Comparator Unvaccinated	139	0	2	1438.84 (174.25, 5197.62)	1438.85 (359.87, 5752.94)	reference	reference	reference	reference
Subgroup: HIV− and PrEP Use									
Fully Vaccinated	37	37.00	0	0.00 (0.00, 0.00)	0.00 (0.00, 0.00)	−2054.79 (−4371.83, 262.24)	−15,721.02 (−32,312.44, 870.40)	NR	NR
Comparator Unvaccinated	51	32.98	3	2054.8 (423.75, 6004.98)	15,721.02 (5471.94, 45,166.87)	reference	reference	reference	reference
Subgroup: HIV− and No PrEP Use									
Fully Vaccinated	20	20.00	0	0.00 (0.00, 0.00)	0.00 (0.00, 0.00)	0.00 (–, –)	0.00 (–, –)	0% (−62%, 38%)	0% (−85%, 46%)
Comparator Unvaccinated	26	19.14	0	0.00 (0.00, 0.00)	0.00 (0.00, 0.00)	reference	reference	reference	reference

NR = not reportable given extremely large variance reported via corresponding risk difference; ^a.^ Subjects in the vaccinated group were coarsened exact matched with 5 subjects who were unvaccinated at the time of match. Subgroups were then determined based on HIV status and ART and PrEP use, noting that the subgroups are not mutually exclusive as may include those with HIV− status and PrEP use and later become HIV+. ^b.^ Mpox was defined as ≥1 ICD-10-CM diagnosis code or laboratory record of mpox PCR test result of “DETECTED”. To ascertain mpox outcome, follow-up began on the index date (14th day after the second MVA–BN dose) and ended on the earliest occurrence of the outcome of interest, disenrollment, death, or end of data availability. ^c.^ Although balance was achieved using 1:1 PS matching, 18 fully vaccinated subjects (>10%) were dropped; thus, SMR weighting was applied assigning vaccinated patients a weight of 1 and unvaccinated subjects a weight of PS/(1 − PS) to create a pseudo-population of weighted unvaccinated comparator subjects.

**Table 3:** Vaccine effectiveness in adult men who have sex with men (MSM) and transgender women, sensitivity analyses ^a^.

	Total No. of Subjects	PS-Adjusted No. of Subjects	Total Subjects with an Mpox Event ^b^	Risk per 100,000 Persons (95% CI)		Risk Difference per 100,000 Persons (95% CI)		Vaccine Effectiveness (95% CI)	
				Crude	Adjusted ^c^	Crude	Adjusted ^c^	Crude	Adjusted ^c^
Sensitivity Analysis: Any Dose (At least one MVA–BN dose)									
Vaccinated	947	908	20	6019.00 (4558.74, 7798.32)	2202.64 (1427.28, 3399.21)	−368.31 (−1410.73, 674.10)	−3854.63 (−5678.38, −2030.87)	14% (−36%, 46%)	64% (40%, 78%)
Comparator Unvaccinated	477	908	55	1048.22 (340.35, 2446.19)	6057.27 (4687.15, 7827.90)	reference	reference	reference	reference
Sensitivity Analysis: Single Dose (One MVA–BN dose only)									
Vaccinated	788	751	13	6008.58 (3992.67, 8684.08)	1731.03 (1009.17, 2969.23)	−564.64 (−1636.11, 506.83)	−3994.67 (−5902.78, −2086.57)	23% (−32%, 55%)	70% (44%, 84%)
Comparator Unvaccinated	3940	751	43	701.75 (569.64, 4094.15)	5725.70 (4281.78, 7656.54)	reference	reference	reference	reference
Sensitivity Analysis: Assumed Prior Smallpox Vaccination (One MVA–BN dose only and age >50 years)									
Vaccinated	215	195	5	8088.24 (4037.62, 14,472.09)	2564.10 (1074.59, 6118.28)	1108.35 (−1019.99, 3236.70)	0.00 (−3153.59, 3153.59)	−91% (−432%, 31%)	0% (−242%, 71%)
Comparator Unvaccinated	1069	195	5	0.00 (0.00, 0.00)	2564.10 (1074.59, 6118.28)	reference	reference	reference	reference

^a.^ Subjects in the vaccinated group were coarsened exact matched with 5 subjects who were unvaccinated at the time of match. ^b.^ Mpox was defined as ≥1 ICD-10-CM diagnosis code or laboratory record of mpox PCR test result of “DETECTED”. To ascertain mpox outcome, follow-up began on the index date (14th day after the second MVA–BN dose) and ended on the earliest occurrence of the outcome of interest, disenrollment, death, or end of data availability. ^c.^ Subjects were 1:1 propensity score matched. Prior to propensity score matching, there were 947 subjects who received at least 1 dose of the MVA–BN vaccine, 788 subjects who received at least 1 dose of the MVA–BN vaccine, and 215 subjects aged >50 years who received 1 MVA–BN vaccine only.

**Table 4:** Baseline characteristics of adult men who have sex with men (MSM) and transgender women, prior to and after propensity score adjustment via 1:1 matching.

Variable	Prior to PS Matching ^a^			After PS Matching ^b^		
	Vaccinated	Comparator Unvaccinated	ASD (Not Matched)	Vaccinated	Comparator Unvaccinated	ASD (Matched)
Number of subjects	947	4735	-	915	915	-
Age on index date			0.000			0.032
18–25 years	70 (7.4%)	350 (7.4%)	-	65 (7.1%)	60 (6.6%)	-
26–35 years	309 (32.6%)	1545 (32.6%)	-	296 (32.3%)	288 (31.5%)	-
36–45 years	247 (26.1%)	1235 (26.1%)	-	239 (26.1%)	246 (26.9%)	-
>45 years	321 (33.9%)	1605 (33.9%)	-	315 (34.4%)	321 (35.1%)	-
US Region on index date			0.000			0.037
Northeast	153 (16.2%)	765 (16.2%)	-	148 (16.2%)	157 (17.2%)	-
Midwest	143 (15.1%)	715 (15.1%)	-	135 (14.8%)	126 (13.8%)	-
South	262 (27.7%)	1310 (27.7%)	-	260 (28.4%)	256 (28.0%)	-
West	389 (41.1%)	1945 (41.1%)	-	372 (40.7%)	376 (41.1%)	-
Insurance Provider on index date			0.000			0.009
Commercial	546 (57.7%)	2730 (57.7%)	-	521 (56.9%)	517 (56.5%)	-
Non-Commercial	401 (42.3%)	2005 (42.3%)	-	394 (43.1%)	398 (43.5%)	-
Race/Ethnicity on index date			0.130			0.075
White	240 (25.3%)	1998 (42.2%)	-	240 (26.2%)	223 (24.4%)	-
Black	140 (14.8%)	940 (19.9%)	-	140 (15.3%)	150 (16.4%)	-
Hispanic	113 (11.9%)	775 (16.4%)	-	113 (12.3%)	123 (13.4%)	-
Asian	18 (1.9%)	165 (3.5%)	-	18 (2.0%)	16 (1.7%)	-
Other	36 (3.8%)	367 (7.8%)	-	36 (3.9%)	35 (3.8%)	-
Missing	400 (42.2%)	490 (10.3%)	-	368 (40.2%)	368 (40.2%)	-
HIV+ during baseline	564 (59.6%)	2690 (56.8%)	0.056	554 (60.5%)	587 (64.2%)	0.074
PrEP use during baseline	281 (29.7%)	1052 (22.2%)	**0.171**	264 (28.9%)	239 (26.1%)	0.061
Time from first PrEP in baseline to index date in days			0.044			0.097
mean (sd)	263.96 (161.87)	271.14 (162.59)		270.44 (161.59)	254.46 (166.94)	
median (IQR)	233.00 [136.00, 442.00]	255.00 [137.00, 427.75]		240.50 [139.00, 450.25]	225.00 [104.00, 421.00]	
STI during baseline ^c^	45 (4.8%)	227 (4.8%)	0.002	43 (4.7%)	38 (4.2%)	0.027
Evidence of (non-HIV) autoimmune disorders or immunocompromised conditions during baseline ^d^	211 (22.3%)	1051 (22.2%)	0.002	205 (22.4%)	216 (23.6%)	0.029
Comorbidities during baseline ^e^	260 (27.5%)	1525 (32.2%)	**0.104**	253 (27.7%)	260 (28.4%)	0.017
History of AESI during baseline						
Myocarditis	0 (0.0%)	2 (0.0%)	0.029	0 (0.0%)	1 (0.1%)	0.047
Pericarditis	2 (0.2%)	6 (0.1%)	0.021	2 (0.2%)	3 (0.3%)	0.021
Encephalitis	0 (0.0%)	4 (0.1%)	0.041	0 (0.0%)	0 (0.0%)	-
Anaphylaxis	1 (0.1%)	3 (0.1%)	0.015	1 (0.1%)	1 (0.1%)	0.000

AESIs = adverse events of special interest; ASD = absolute standardized differences; HIV = human immunodeficiency virus; IQR = interquartile range; MSM = men who have sex with men; PrEP = pre-exposure prophylaxis; sd = standard deviation; STI = sexually transmitted infections; US = United States. Index date for the vaccinated group = the date of the qualifying MVA–BN. Baseline period = Start of data (1 April 2022) to 1 day before Index Date. ^a.^ Subjects in the vaccinated group (N = 947) were coarsened exact matched with 5 unvaccinated comparator subjects who were unvaccinated at the time of the match on calendar date, age, region, and insurance provider (N = 4735). ^b.^ Vaccinated subjects were subsequently 1:1 propensity score matched to unvaccinated comparator subjects to select a comparable matched sample of the analytic cohort. ^c.^ Sexually transmitted infections (STI) were chlamydia, gonorrhea, syphilis, hepatitis B. ^d.^ Autoimmune disease, immunodeficiency, inhaled corticosteroids, immunomodulating medications, and immunotherapy. ^e.^ Cancer, diabetes, atopic dermatitis (neurodermatitis), and rheumatological, hematological, chronic cardiovascular, chronic lung, chronic kidney, and chronic liver disease. Note: ASDs ≥ 0.10 have been depicted in bold text.

**Table 5:** Vaccine AESIs among men who have sex with men (MSM) and transgender women, among a PS-matched cohort of 915 vaccinated subjects and 915 comparator unvaccinated subjects ^a^.

	Total No. of Subjects with an AESI Event ^b^	Risk per 100,000 Persons (95% CI)	Risk Difference per 100,000 Persons (95% CI)	Total No. of Subjects with an AESI Event ^b^	Risk per 100,000 Persons (95% CI)	Risk Difference per 100,000 Persons (95% CI)
	**Anaphylaxis**					
	**Primary 1-day risk window**			**Sensitivity 3-day risk window**		
Vaccinated	0	-	-	0	-	-
Comparator Unvaccinated	0	-	reference	0	-	reference
	**Myocarditis**					
	**Primary 14-day risk window**			**Sensitivity 28-day risk window**		
Vaccinated	0	-	-	0	-	-
Comparator Unvaccinated	0	-	reference	0	-	reference
	**Pericarditis**					
	**Primary 14-day risk window**			**Sensitivity 28-day risk window**		
Vaccinated	0	-	-	1	109.29 (15.38, 776.69)	109.29 (−105.03, 323.61)
Comparator Unvaccinated	0	-	reference	0	0.00 (0.00, 0.00)	reference
	**Encephalitis (Primary Definition)**					
	**Primary 28-day risk window**			**Sensitivity 42-day risk window**		
Vaccinated	0	-	-	0	-	-
Comparator Unvaccinated	0	-	reference	0	-	reference
	**Encephalitis (Broader Definition)**					
	**Primary 28-day risk window**			**Sensitivity 42-day risk window**		
Vaccinated	0	0.00 (0.00, 0.00)	−109.29 (−323.61, 105.03)	0	0.00 (0.00, 0.00)	−109.29 (−323.61, 105.03)
Comparator Unvaccinated	1	109.29 (15.38, 776.69)	reference	1	109.29 (15.38, 776.69)	reference

^a.^ Subjects in the vaccinated group were coarsened exact matched with 5 subjects who were unvaccinated at the time of match. Vaccinated subjects were subsequently 1:1 propensity score matched to unvaccinated comparator subjects to select a comparable matched sample of the analytic cohort. Prior to the PS adjustment, there were 947 subjects in the vaccinated group and 4735 subjects in the comparator unvaccinated group. ^b.^ AESIs = Adverse Events of Special Interest, which were selected in line with the outcomes classified as important potential risks for MVA–BN in the EU Risk Management Plan. Follow-up began on the index date (first MVA–BN dose after meeting eligibility criteria between 1 August 2022 and 30 September 2022) and ended on the earliest occurrence of the outcome of interest, disenrollment, death, end of data availability, or end of the AESI-specific risk window. Additionally, unvaccinated subjects were censored upon cross-over to the vaccinated group upon receiving the first dose of MVA–BN vaccination. Anaphylaxis: ICD-10 T78.2, T78.2XXS, T80.5, T88.6, T80.52XA, T78.2XXA, T78.08XA; Encephalitis—Primary: ICD-10 G04.00, G04.01, G04.02, G04.81, G04.90, G05.3; Encephalitis—Broad: Primary + non-specific ICD-10 G36.9, G37.3, G37.9; Myocarditis: ICD-10 B33.22, I40.1, I40.9, I40.8, I40.9, I41, I51.4; Pericarditis: ICD-10 B33.23, I30.0, I30.1, I30.8, I30.9, I32.

## Data Availability

Restrictions apply to the availability of these data. Data were licensed from HealthVerity. Access to the data requires purchasing from the data vendor directly.

## Supplementary Materials

The following supporting information can be downloaded at: https://www.mdpi.com/article/10.3390/vaccines12060651/s1, Table S1: MSM Criteria; Table S2: Baseline characteristics of adult men who have sex with men (MSM) and transgender women, by HIV and Treatment status prior to SMR weighting; Table S3: Baseline characteristics of adult men who have sex with men (MSM) and transgender women, by HIV and Treatment status after SMR weighting.
