# Supplementary material for: Effectiveness and Safety of the MVA–BN Vaccine against Mpox in At-Risk Individuals in the United States (USMVAc)
Source: Vaccines (Basel). 2024 Jun 11;12(6):651. doi: 10.3390/vaccines12060651 (PMC11209565; doi:10.3390/vaccines12060651)
Supplement: Supplementary file 1 [file vaccines-12-00651-s001.zip › vaccines-2962823-supplementary.pdf]

Table S1. MSM Criteria.

	MSM Criteria
1.	Male according to sex assigned at birth
2.	Age 18 years or older between April 1, 2021, and December 31, 2022
3.	Identify as MSM or transgender woman according to the following proxy between 1 April 2021 and 31 December 2022 if they met at least 1 of the following criteria: ● Evidence related to High-Risk Sexual Behavior (HRSB) defined by International Classification of Diseases, Tenth Revision, Clinical Modification (ICD-10-CM) codes, Z72.51, Z72.52, or Z72.53; OR ● Evidence of HIV infection by ICD-10 codes: B20 or Z21; OR ● Evidence of HIV PrEP therapy with any of the following National Drug Codes (NDC): 6195807041, 6195807051, 6195807031, 6195807011, 6362975813, 6729612373, 6729612374, 6362975811, 6362975812, 6195820051, 6195820021, 6195820022, 4970226423, 4970224015, 4970225315, 4970224813; or Current Procedural Terminology (CPT); J0741, J0739 AND No history of substance use disorder within the study period identified by the following ICD-10-CM; F11*, F12*, F13*, F14*, F15*, F16*, F18, F19* (to accurately capture consistent users of PrEP and the population for whom PrEP is indicated to prevent HIV infection among MSM and transgender women)

Table S2. Baseline characteristics of adult men who have sex with men (MSM) and transgender women, by HIV and Treatment status prior to SMR weighting [a.]

Prior to SMR Weighting												
	**HIV+ and Antiretroviral Use**			**HIV+ and No Antiretroviral Use**			**HIV- and PrEP Use**			**HIV- and No PrEP Use**		
**Variable**	**Fully Vaccinated**	**Comparator Unvaccinated**	**ASD [b] (not weighted)**	**Fully Vaccinated**	**Comparator Unvaccinated**	**ASD [b] (not weighted)**	**Fully Vaccinated**	**Comparator Unvaccinated**	**ASD [b] (not weighted)**	**Fully Vaccinated**	**Comparator Unvaccinated**	**ASD [b] (not weighted)**
Number of subjects	104	316	-	0	139	-	37	146	-	20	209	-
Age on index date												
...18-25 years	7 (6.7%)	9 (2.8%)	**0.183**	0 (0.0%)	3 (2.2%)	-	1 (2.7%)	6 (4.1%)	0.078	2 (10.0%)	32 (15.3%)	**0.160**
...26-35 years	28 (26.9%)	69 (21.8%)	**0.119**	0 (0.0%)	29 (20.9%)	-	15 (40.5%)	66 (45.2%)	0.094	9 (45.0%)	95 (45.5%)	0.009
...36-45 years	28 (26.9%)	99 (31.3%)	0.097	0 (0.0%)	43 (30.9%)	-	11 (29.7%)	35 (24.0%)	**0.130**	5 (25.0%)	43 (20.6%)	**0.106**
...>45 years	41 (39.4%)	139 (44.0%)	0.093	0 (0.0%)	64 (46.0%)	-	10 (27.0%)	39 (26.7%)	0.007	4 (20.0%)	39 (18.7%)	0.034
Region in the US on index date												
...Northeast	15 (14.4%)	45 (14.2%)	0.005	0 (0.0%)	32 (23.0%)	-	3 (8.1%)	18 (12.3%)	**0.140**	7 (35.0%)	35 (16.7%)	**0.426**
...Midwest	9 (8.7%)	42 (13.3%)	**0.149**	0 (0.0%)	18 (12.9%)	-	9 (24.3%)	19 (13.0%)	**0.293**	7 (35.0%)	46 (22.0%)	**0.291**
...South	32 (30.8%)	79 (25.0%)	**0.129**	0 (0.0%)	30 (21.6%)	-	7 (18.9%)	26 (17.8%)	0.029	0 (0.0%)	58 (27.8%)	**0.877**
...West	48 (46.2%)	150 (47.5%)	0.026	0 (0.0%)	59 (42.4%)	-	18 (48.6%)	83 (56.8%)	**0.165**	6 (30.0%)	70 (33.5%)	0.075
Insurance Provider on index date												
...Commercial	53 (51.0%)	181 (57.3%)	**0.127**	0 (0.0%)	52 (37.4%)	-	25 (67.6%)	111 (76.0%)	**0.189**	14 (70.0%)	123 (58.9%)	**0.235**
...Non-Commercial	51 (49.0%)	135 (42.7%)	**0.127**	0 (0.0%)	87 (62.6%)	-	12 (32.4%)	35 (24.0%)	**0.189**	6 (30.0%)	86 (41.1%)	**0.235**
Race/Ethnicity on index date												
...White	26 (25.0%)	133 (42.1%)	**0.368**	0 (0.0%)	54 (38.8%)	-	14 (37.8%)	82 (56.2%)	**0.374**	9 (45.0%)	102 (48.8%)	0.076

...Race other than white [c]	36 (34.6%)	149 (47.2%)	**0.257**	0 (0.0%)	78 (56.1%)	-	9 (24.3%)	54 (37.0%)	**0.277**	2 (10.0%)	100 (47.8%)	**0.919**
...Missing	42 (40.4%)	34 (10.8%)	**0.722**	0 (0.0%)	7 (5.0%)	-	14 (37.8%)	10 (6.8%)	**0.801**	9 (45.0%)	7 (3.3%)	**1.113**
HIV+ during baseline	104 (100.0%)	316 (100.0%)	-	0 (0.0%)	139 (100.0%)	-	0 (0.0%)	0 (0.0%)	-	0 (0.0%)	0 (0.0%)	-
PrEP use during baseline	11 (10.6%)	32 (10.1%)	0.015	0 (0.0%)	0 (0.0%)	-	37 (100.0%)	146 (100.0%)	-	0 (0.0%)	0 (0.0%)	-
Time from first PrEP in baseline to index date												
...0-6 months	4 (3.8%)	9 (2.8%)	0.056	0 (0.0%)	0 (0.0%)	-	15 (40.5%)	42 (28.8%)	**0.249**	0 (0.0%)	0 (0.0%)	-
...6 months - 1 year	3 (2.9%)	7 (2.2%)	0.043	0 (0.0%)	0 (0.0%)	-	9 (24.3%)	50 (34.2%)	**0.219**	0 (0.0%)	0 (0.0%)	-
…≥ 1 year	4 (3.8%)	16 (5.1%)	0.059	0 (0.0%)	0 (0.0%)	-	13 (35.1%)	54 (37.0%)	0.039	0 (0.0%)	0 (0.0%)	-
...No PrEP in baseline	93 (89.4%)	284 (89.9%)	0.015	0 (0.0%)	139 (100.0%)	-	0 (0.0%)	0 (0.0%)	-	20 (100.0%)	209 (100.0%)	-
History of STI during baseline [d]	7 (6.7%)	15 (4.7%)	0.085	0 (0.0%)	9 (6.5%)	-	0 (0.0%)	9 (6.2%)	**0.363**	1 (5.0%)	7 (3.3%)	0.083
Evidence of autoimmune disorders or immunocompromised conditions (non-HIV) during baseline [e]	35 (33.7%)	105 (33.2%)	0.009	0 (0.0%)	19 (13.7%)	-	9 (24.3%)	46 (31.5%)	**0.161**	2 (10.0%)	42 (20.1%)	**0.285**
Comorbidities during baseline [f]	34 (32.7%)	117 (37.0%)	0.091	0 (0.0%)	67 (48.2%)	-	5 (13.5%)	35 (24.0%)	**0.270**	5 (25.0%)	45 (21.5%)	0.082

PrEP = pre-exposure prophylaxis. Index date for the fully vaccinated group = the 14th day after the date of the second dose of MVA-BN between 1 August 2022 and 30 September 2022. Baseline period = Start of data (1 April 2022) to 1 day before Index Date. a. Subjects in the fully vaccinated group (N=163) were coarsened exact matched with up to 5 unvaccinated comparator subjects on calendar date, age, region, and insurance provider N=815). Initially, 1:1 PS matching was attempted. Although balance was achieved using 1:1 matching, 18 fully vaccinated subjects were dropped (>10% of the exposed group); thus, SMR weighting was applied as an alternative to the PS modeling approach. In the adjusted models, comparator unvaccinated subjects were SMR-weighted using the calculated propensity score of those who received a vaccination, assigning a weight of PS / (1 - PS) to create a pseudo-population of weighted unvaccinated comparator subjects, and vaccinated patients received a weight of 1 remaining the same for crude and adjusted. b. ASD = absolute standardized differences; ASDs ≥0.10 have been depicted in bold text. c. Includes subjects with a race recorded as Black, Hispanic, Asian, or Other. d. STI = sexually transmitted infection, and includes the following conditions: chlamydia, gonorrhea, syphilis, hepatitis B. e. Evidence of autoimmune disorders or immunocompromised conditions (non-HIV) includes the following conditions: autoimmune disease, immunodeficiency, inhaled/dermatological corticosteroid use, immunomodulating medication use, and immunotherapy. f. Comorbidities include the following conditions: rheumatological disease, cancer, hematological disease, chronic cardiovascular disease (i.e., heart failure, coronary artery disease, cardiomyopathies), chronic lung disease, chronic kidney disease, chronic liver disease, type I or II diabetes, and atopic dermatitis (neurodermatitis).

Table S3. Baseline characteristics of adult men who have sex with men (MSM) and transgender women by HIV and Treatment status after SMR weighting.

	HIV+ and Antiretroviral Use			*HIV+ and No Antiretroviral Use*			*HIV- and PrEP Use*			*HIV- and No PrEP Use*		
Variable	*Fully Vaccinated*	*Comparator Unvaccinated a*	*ASD b*	*Fully Vaccinated*	*Comparator Unvaccinated a*	*ASD b*	*Fully Vaccinated*	*Comparator Unvaccinated a*	*ASD b*	*Fully Vaccinated*	*Comparator Unvaccinated a*	*ASD b*
Effective sample size	*104*	*134.00*	*-*	*0*	*139.00*	*-*	*37*	*51.00*	*-*	*20*	*26.00*	*-*
Sum of weights	*104*	*107.52*	*-*	*0*	*0.00*	*-*	*37*	*32.98*	*-*	*20*	*19.14*	*-*
Age on index date												
...18-35 years	*35.0 (33.7%)*	*41.8 (38.9%)*	*0.109*	*0.0 (-%)*	*0.0 (23.0%)*	*-*	*16.0 (43.2%)*	*14.2 (43.1%)*	*0.004*	*11.0 (55.0%)*	*9.3 (48.5%)*	*0.131*
...36-45 years	*28.0 (26.9%)*	*24.2 (22.6%)*	*0.102*	*0.0 (-%)*	*0.0 (30.9%)*	*-*	*11.0 (29.7%)*	*9.2 (28.0%)*	*0.037*	*5.0 (25.0%)*	*5.1 (26.8%)*	*0.042*

...>45 years	41.0 (39.4%)	41.5 (38.6%)	0.017	0.0 (-%)	0.0 (46.0%)	-	10.0 (27.0%)	9.5 (28.9%)	0.042	4.0 (20.0%)	4.7 (24.7%)	0.113
Region in the US on index date												
...Northeast	15.0 (14.4%)	16.6 (15.5%)	0.029	0.0 (-%)	0.0 (23.0%)	-	3.0 (8.1%)	2.1 (6.4%)	0.067	7.0 (35.0%)	6.4 (33.2%)	0.037
...Midwest	9.0 (8.6%)	9.4 (8.7%)	0.003	0.0 (-%)	0.0 (13.0%)	-	9.0 (24.3%)	5.7 (17.4%)	0.171	7.0 (35.0%)	5.7 (30.0%)	0.108
...South	32.0 (30.8%)	27.4 (25.5%)	0.119	0.0 (-%)	0.0 (21.6%)	-	7.0 (18.9%)	10.1 (30.8%)	0.277	0.0 (0.0%)	0.0 (0.0%)	0.000
...West	48.0 (46.2%)	54.1 (50.4%)	0.084	0.0 (-%)	0.0 (42.4%)	-	18.0 (48.7%)	15.0 (45.5%)	0.064	6.0 (30.0%)	7.0 (36.8%)	0.145
Insurance Provider on index date												
...Commercial	53.0 (51.0%)	48.6 (45.2%)	0.116	0.0 (-%)	0.0 (37.4%)	-	25.0 (67.6%)	22.5 (68.2%)	0.013	14.0 (70.0%)	13.6 (71.3%)	0.029
...Non-Commercial	51.0 (49.0%)	59.0 (54.8%)	0.116	0.0 (-%)	0.0 (62.6%)	-	12.0 (32.4%)	10.5 (31.8%)	0.013	6.0 (30.0%)	5.5 (28.7%)	0.029
Race/Ethnicity on index date												
...White	26.0 (25.0%)	25.0 (23.3%)	0.040	0.0 (-%)	0.0 (38.9%)	-	14.0 (37.8%)	13.8 (41.9%)	0.084	9.0 (45.0%)	9.3 (48.4%)	0.068
...Race other than white c	36.0 (34.6%)	36.3 (33.8%)	0.018	0.0 (-%)	0.0 (56.1%)	-	9.0 (24.3%)	8.6 (26.0%)	0.038	2.0 (10.0%)	2.0 (10.5%)	0.016
...Missing	42.0 (40.4%)	46.2 (43.0%)	0.052	0.0 (-%)	0.0 (5.0%)	-	14.0 (37.8%)	10.6 (32.1%)	0.121	9.0 (45.0%)	7.9 (41.1%)	0.078
HIV+ during baseline	104.0 (100.0%)	107.5 (100.0%)	-	0.0 (-%)	0.0 (100.0%)	-	0.0 (0.0%)	0.0 (0.0%)	-	0.0 (0.0%)	0.0 (0.0%)	-
PrEP use during baseline	11.0 (10.6%)	11.2 (10.4%)	0.006	0.0 (-%)	0.0 (0.0%)	-	37.0 (100.0%)	33.0 (100.0%)	-	0.0 (0.0%)	0.0 (0.0%)	-
Time from first PrEP in baseline to index date												
...0-6 months	4.0 (3.9%)	4.4 (4.1%)	0.010	0.0 (-%)	0.0 (0.0%)	-	15.0 (40.5%)	11.1 (33.6%)	0.145	0.0 (0.0%)	0.0 (0.0%)	-
...6 months - 1 year	3.0 (2.9%)	2.7 (2.5%)	0.024	0.0 (-%)	0.0 (0.0%)	-	9.0 (24.3%)	9.0 (27.4%)	0.070	0.0 (0.0%)	0.0 (0.0%)	-
…≥ 1 year	4.0 (3.9%)	4.2 (3.9%)	0.001	0.0 (-%)	0.0 (0.0%)	-	13.0 (35.1%)	12.9 (39.1%)	0.081	0.0 (0.0%)	0.0 (0.0%)	-
...No PrEP in baseline	93.0 (89.4%)	96.3 (89.6%)	0.006	0.0 (-%)	0.0 (100.0%)	-	0.0 (0.0%)	0.0 (0.0%)	-	20.0 (100.0%)	19.1 (100.0%)	-
History of STI during baseline d	7.0 (6.7%)	8.3 (7.8%)	0.039	0.0 (-%)	0.0 (6.5%)	-	0.0 (0.0%)	0.0 (0.0%)	0.000	1.0 (5.0%)	0.5 (2.6%)	0.124
Evidence of autoimmune disorders or immunocompromised conditions (non-HIV) during baseline e	35.0 (33.7%)	32.3 (30.0%)	0.078	0.0 (-%)	0.0 (13.7%)	-	9.0 (24.3%)	10.6 (32.2%)	0.175	2.0 (10.0%)	1.5 (7.9%)	0.073
Comorbidities during baseline f	34.0 (32.7%)	33.2 (30.9%)	0.038	0.0 (-%)	0.0 (48.2%)	-	5.0 (13.5%)	5.5 (16.6%)	0.087	5.0 (25.0%)	5.2 (27.0%)	0.046

PrEP = pre-exposure prophylaxis. Index date for the fully vaccinated group = the 14th day after the date of the second dose of MVA-BN between 1 August 2022 and 30 September 2022. Baseline period = Start of data (1 April 2022) to 1 day before Index Date. a. Subjects in the fully vaccinated group (N=163) were coarsened exact matched with up to 5 unvaccinated comparator subjects on calendar date, age, region, and insurance provider N=815). Initially, 1:1 PS matching was attempted. Although balance was achieved using 1:1 matching, 18 fully vaccinated subjects were dropped (>10% of the exposed group); thus, SMR weighting was applied as an alternative to the PS modeling approach. In the adjusted models, comparator unvaccinated subjects were SMR-weighted using the calculated propensity score of those who received a vaccination, assigning a weight of PS / (1 - PS) to create a pseudo-population of weighted unvaccinated comparator subjects, and vaccinated patients received a weight of 1 remaining the same for crude and adjusted. b. ASD= absolute standardized differences; ASDs ≥0.10 have been depicted in bold text. c. Includes subjects with a race recorded as Black, Hispanic, Asian, or Other. d. STI = sexually transmitted infection, and includes the following conditions: chlamydia, gonorrhea, syphilis, hepatitis B. e. Evidence of autoimmune disorders or immunocompromised conditions (non-HIV) includes the following conditions: autoimmune disease, immunodeficiency, inhaled/dermatological corticosteroid use, immunomodulating medication use, and immunotherapy. f. Comorbidities include the following conditions: rheumatological disease, cancer, hematological disease, chronic cardiovascular disease (i.e., heart failure, coronary artery disease, cardiomyopathies), chronic lung disease, chronic kidney disease, chronic liver disease, type I or II diabetes, and atopic dermatitis (neurodermatitis).
